# Brown Spider Venom Phospholipases D: From Potent Molecules Involved in Pathogenesis of Brown Spider Bites to Molecular Tools for Studying Ectosomes, Ectocytosis, and Its Applications

**DOI:** 10.3390/toxins17020070

**Published:** 2025-02-05

**Authors:** Ana Carolina Martins Wille, Mariana Izabele Machado, Samira Hajjar Souza, Hanna Câmara da Justa, Maria Eduarda de Fraga-Ferreira, Eloise de Souza Mello, Luiza Helena Gremski, Silvio Sanches Veiga

**Affiliations:** 1 Department of Structural, Molecular Biology and Genetics, State University of Ponta Grossa (UEPG), Ponta Grossa 84030-900, Brazil; anacarolina.wille@yahoo.com.br; 2 Department of Cell Biology, Federal University of Paraná (UFPR), Curitiba 81530-900, Brazil; mariana.machado1@ufpr.br (M.I.M.); samirahajjar@ufpr.br (S.H.S.); hannajusta@gmail.com (H.C.d.J.); mariafraga@ufpr.br (M.E.d.F.-F.); eloisemello@ufpr.br (E.d.S.M.); luizagremski@ufpr.br (L.H.G.)

**Keywords:** brown spider venom phospholipases D, biochemical properties, plasma membrane, ectosomes, signalosomes

## Abstract

Accidents caused by *Loxosceles* spiders, commonly known as brown spiders, are frequent in warm and temperate regions worldwide, with a higher prevalence in South America and the southern United States. In the venoms of species clinically associated with accidents, phospholipases D (PLDs) are the most expressed toxins. This classification is based on the toxins’ ability to cleave various phospholipids, with a preference for sphingomyelin. Studies using purified PLDs have demonstrated that these enzymes cleave phospholipids from cells, producing derivatives that can activate leukocytes. A dysregulated inflammatory response is the primary effect following envenomation, leading to dermonecrosis, which is histopathologically characterized by aseptic coagulative necrosis—a key feature of envenomation. Although advances in understanding the structure–function relationship of enzymes have been achieved through molecular biology, heterologous expression, site-directed mutations, crystallography, and bioinformatic analyses—describing PLDs in the venoms of various species and highlighting the conservation of amino acid residues involved in catalysis, substrate binding, and magnesium stabilization—little is known about the cellular biology of these PLDs. Studies have shown that the treatment of various cells with recombinant PLDs stimulates the formation of ectosomes and ectocytosis, events that initiate a cascade of intracellular signaling in PLD-binding cells and lead to the release of extracellular microvesicles. These microvesicles may act as signalosomes for other target cells, thereby triggering an inflammatory response and dermonecrosis. In this review, we will discuss the biochemical properties of PLDs, the target cells that bind to them, and the ectocytosis-dependent pathophysiology of envenoming.

## 1. Introduction

Accidents caused by brown spiders (genus *Loxosceles*) are reported on several continents, but they are most prevalent in warm regions, such as South America and the southern United States [1,2,3]. Loxoscelism, the term used to describe clinical signs and symptoms, is characterized by a lesion at the bite site and surrounding areas, which can progress to dermonecrosis with gravitational spreading (a hallmark of these accidents). In some cases, systemic involvement may occur, leading to complications such as intravascular hemolysis, hemolytic anemia, and acute renal failure [1,2,3]. Although there are over a hundred species of *Loxosceles* distributed across all five continents [4], the venoms described so far are highly conserved, consisting primarily of proteins and peptides ranging between 3 and 45 kDa [3,5,6]. During bites, it is believed that only small amounts of venom are injected into the skin, typically not exceeding a few microliters and containing about 50 to 100 micrograms of crude venom. This highlights the high biological activity of these venoms, especially when compared to snake venoms, which often involve the injection of several milliliters of crude venom containing milligrams of proteins [7,8]. Several proteins have been identified in the crude venoms of *Loxosceles*, including metalloproteases, serine proteases, hyaluronidases, serpin-like serine protease inhibitors, allergenic factors, TCTP, low-molecular-mass peptides characterized as Knottins, and phospholipases D (PLDs) [3,9,10,11,12]. Among the toxins in *Loxosceles* venoms, PLDs are the most studied and widely expressed, with the most extensive information available [2,8,12,13]. This toxin, which consists of a broad and conserved family of isoforms/homologues across different species’ venoms, plays a key role in the pathophysiology of both cutaneous and systemic loxoscelism [1,2,3,8]. Under laboratory conditions, PLDs from various species around the world reproduce the signs and symptoms caused by crude venoms, including skin necrosis, a deep inflammatory response at the inoculation site, hemolysis, and cytotoxicity in renal cell lines, as well as nephrotoxicity in vivo, demonstrating their role in loxoscelism [2,3,8]. Given their involvement in the pathophysiology of loxoscelism, PLDs can be used as tools for immunotherapy (such as antibody-based *Loxosceles* venom therapy), the development of pharmacological treatments, and/or laboratory diagnosis [14,15,16]. Additionally, since PLDs target various cells and cell components, particularly cell membranes and phospholipids, they have the potential to serve as valuable biotools in the biological sciences. In this review, we will discuss published data on the biochemical, biological, and biotechnological properties of PLDs, which underscore the importance of research on these toxins. This research may advance our understanding of cell biology, based on toxin interactions with the plasma membrane, ectosome formation, and ectocytosis.

## 2. Biochemical, Molecular, and Structural Properties of Brown Spider Venom Phospholipases D

As mentioned earlier, PLDs are the most extensively studied and widely expressed toxins in *Loxosceles* venoms. The first data on these toxins were reported in 1978 [17], revealing an enzyme in *L. reclusa* venom that degrades sphingomyelin (a component of the extracellular monolayer of cell membranes). This enzyme produces ceramide-1-phosphate and choline, leading to the hemolysis of sheep and human red blood cells. The authors named it sphingomyelinase D due to its action on sphingomyelin, which generates choline. In the following years, it was demonstrated that, in addition to sphingomyelin, these enzymes also act on various phospholipids in cell membranes. Studies of recombinant PLDs from various *Loxosceles* species have shown that these enzymes can degrade several phospholipids, including sphingomyelin, lysophosphatidylcholine, lysophosphatidylethanolamine, lysophosphatidylserine, lysophosphatidylinositol, cyclic phosphatidic acid, lyso-PAF, and other substrates [18,19,20]. Due to this broad range of activity, the nomenclature for phospholipases D was proposed [18]. However, since these PLDs act preferentially on sphingomyelin, the term sphingomyelinase D is also used in the literature to refer to these enzymes [2,8]. As mentioned, the small volume and low protein concentration of *Loxosceles* venoms make the purification of their components a challenging task. Advancements in the field were achieved through molecular biology techniques, such as gene cloning and the expression of recombinant PLDs. By studying species prevalent in South America, such as *L. intermedia*, *L. laeta*, and *L. gaucho*, as well as those in North America, like *L. reclusa*, *L. arizonica*, and *L. deserta*, scientists were able to clone several PLD genes and identify a family of these enzymes in these venoms with both intra- and inter-species variations [2,8,21,22,23]. By using recombinant PLDs, phylogenetic analyses, and crystallography, researchers were able to gather information about this family of toxins. These proteins have 284 to 285 amino acid residues, molecular masses between 30 and 35 kDa, and highly conserved amino acid identities ranging from 55% to 99% [2,8,13,24]. PLDs are composed of a single subunit arranged in a distorted barrel structure, featuring eight parallel beta sheets connected by loop regions to eight alpha helices, and are referred to as Barrel (alpha/beta)8. The molecular organization of PLDs includes three distinct loops: catalytic, flexible, and variable [25,26]. Structural and amino acid analyses revealed two groups of PLDs, classified based on the number of disulfide bridges. These bridges contribute to the molecular structure and stability of the enzyme, as well as to the regulation of the size of the catalytic cleft. One group, which includes PLDs produced by *Loxosceles laeta*, contains a single intramolecular disulfide bridge between residues Cys51 and Cys57. Another group, found in various species, features PLDs with two disulfide bridges: one between residues Cys51 and Cys57, and an additional one between Cys53 and Cys201 [25,26]. Indeed, other amino acid sites appear to influence the activity of these enzymes on phospholipids. Comparative analysis between a Class 1 PLD isoform produced by *L. laeta*, which has a single disulfide bridge, and a Class 2 PLD produced by *L. intermedia*, which has two disulfide bridges, revealed that the *L. laeta* PLD exhibited greater degrading activity on sphingomyelin and lysophosphatidylcholine [27]. Nevertheless, the importance of disulfide bridges was demonstrated through the expression of PLDs with site-directed mutations. A variant of *L. intermedia* PLD was produced with only one disulfide bridge between residues Cys51 and Cys57, and with additional mutations at residues C53A and C201A. This PLD variant showed a significant reduction in its degrading activities on sphingomyelin and lysophosphatidylcholine compared to the wild-type enzyme [2]. Studies based on crystallography of recombinant PLDs from *L. laeta*, *L. intermedia*, and *L. arizonica* [25,26,28,29], along with cloning studies of PLDs from various species’ venoms [2,8], have shown that all described PLDs contain conserved amino acid residues His12, Glu32, Asp34, His47, Asp91, Trp230, Asp233, and Asn252, which are involved in catalysis and/or substrate binding [25,26,29]. Residues His12 and His47 are directly involved in the catalytic activity of PLDs, interacting with substrates to be cleaved. The amino acid residues Glu32, Asp34, and Asp91 play a role in stabilizing and coordinating a magnesium ion in the enzyme, as these enzymes are metalloproteins. The magnesium ion acts in recognizing and binding phospholipid substrates on the cell surface, contributing to chemical stabilization between the enzyme and substrates, and also plays a role in ectosome formation [25,26,29,30]. Additionally, residue Trp230, either alone or in combination with Tyr228 and Tyr229, is involved in binding the hydrophilic choline head through cation–π interactions between choline and the aromatic residues of these amino acids [31,32]. Although crystallographic data and nuclear magnetic resonance analyses have provided significant insights into the mechanism of catalysis and interactions between PLDs and phospholipids, some details of this binary relationship remain unresolved. Two possible catalytic mechanisms for the degradation of phospholipids by PLDs from *Loxosceles* venoms, using sphingomyelin as a prototype substrate, have been proposed. The first mechanism involves a hydrolysis reaction, where residue His47 acts as a nucleophile targeting the phosphodiester bond, releasing choline, while residue His12 participates in the formation of the final product, ceramide-1-phosphate [25,26,29]. The second mechanism proposes a transphosphatidylation reaction, producing cyclic ceramide-1-phosphate, with both His12 and His47 residues, along with the magnesium ion, playing important roles in the catalytic process [19,20,28]. Both hypotheses may be valid and are not mutually exclusive, as they are supported by strong theoretical and experimental evidence. Recombinant isoforms of PLDs with site-directed mutations in key amino acid residues (H12, H47, E32, D34, Y228, Y229, and W230) exhibit only residual phospholipase D and cytotoxic activities compared to the wild type, highlighting the crucial role of these amino acids in enzyme function [2,31,32]. For more details on the structural and molecular characteristics of brown spider PLDs, readers are encouraged to consult the references [8,27,30,33].

## 3. The Role of Phospholipases D in the Toxic Effects Observed Following Accidents

**Figure 1:**
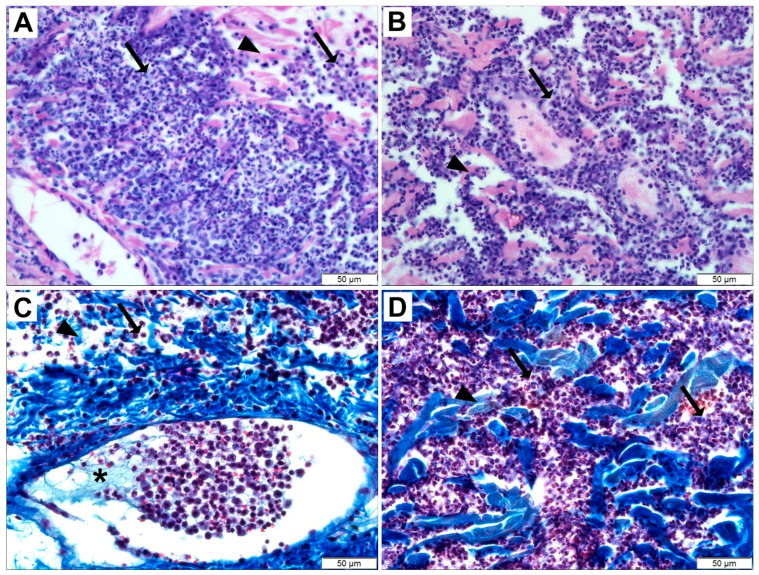
Histopathological analysis of rabbit skin exposed to recombinant PLD from *Loxosceles intermedia* venom. Light microscopy of skin samples collected 24 h after PLD exposure, stained with hematoxylin–eosin (**A**,**B**) or Masson’s Trichrome (**C**,**D**). Results reveal intense inflammation marked by abundant leukocytes in the dermis (black arrows), collagen disorganization indicating edema (black arrowheads), and fibrin network formation with adhered leukocytes within blood vessels (asterisk). For methodological details, see reference [52].

Although *Loxosceles* spiders have a greater incidence in countries or regions with temperate or warm climates such as in South America, southern areas of the United States of America, and Africa, they have been described worldwide [4,8,13]. The main clinical characteristics of loxoscelism typically appear about 4 h after a bite, becoming more pronounced in the following hours and days. These classic signs include skin lesions affecting the epidermis and dermis around the bite site and adjacent areas. They are characterized by tissue necrosis of varying sizes, deep inflammatory infiltrates (Figure 1), localized edema, erythema, hemorrhagic events at the necrotic site, darkened scabs, and the hallmark sign of loxoscelism: necrosis that progresses in a gravitational manner, resembling drops of water moving downward (gravitational spreading of the lesion) [1,3,34]. Although much less frequent, systemic loxoscelism is associated with clinical complications. Systemic manifestations may include intravascular hemolysis, hemolytic anemia, acute renal failure, and thrombocytopenia [1,3,8,34]. PLDs play a fundamental role in the pathophysiology of both cutaneous and systemic loxoscelism. The presence of one or more toxins in the venoms of *Loxosceles* spiders, related to the cutaneous effects observed after bites, has been anticipated since the mid-20th century, when physicians involved in treating victims had postulated the existence of toxins responsible for skin necrosis [35,36]. Research has advanced over the years, and it is now well-established that PLDs are responsible for the necrotic lesions observed after bites. Recombinant isoforms of PLDs from the venoms of various species have significantly contributed to this understanding. When injected into the skin of rabbits, these isoforms caused dermonecrotic lesions with gravitational spreading, as well as blackened scars, edema, hemorrhage, and erythema—signs similar to those observed in experiments with crude venoms and in patients. This further supports the role of these toxins in the pathophysiology of the lesions [2,8]. The involvement of PLDs in necrotic events is also supported by immunological evidence. Polyclonal sera and monoclonal antibodies, produced from animals sensitized with purified PLDs or synthetic peptide sequences from PLDs, have been effective in neutralizing the necrotizing and hemolytic activities of crude venoms from different species, as well as in reducing the noxious effects caused by purified PLDs [37,38,39,40]. Finally, an experimental vaccination protocol using mutated isoforms of recombinant PLDs from venoms of species prevalent in South America successfully protected animals (rabbits and mice) from the necrotizing, edematogenic, hemorrhagic effects, and lethality caused by crude venoms [2,41]. The role of PLDs in systemic loxoscelism is well-supported by laboratory and experimental evidence. Their involvement in intravascular hemolysis and hemolytic anemia—two major clinical manifestations of systemic loxoscelism—is strongly demonstrated by their hemolytic activity on sheep and human red blood cells in vitro, as well as by causing anisocytosis and poikilocytosis in human erythrocytes under culture conditions [42]. Another systemic manifestation of loxoscelism is acute renal failure, which, though less common than hemolytic alterations, is associated with severe complications that can lead to death. Laboratory and experimental data demonstrated that PLDs contribute to kidney injuries observed in patients. This is supported by studies showing that purified recombinant PLDs exert cytotoxic effects on kidney cell lines in culture and bind to their surfaces. Additionally, animals treated with recombinant PLDs exhibit signs of renal failure, including proteinuria, hemoglobinuria, and hematuria. Histopathological analyses of kidneys reveal glomerular edema, tubular necrosis, and proteinaceous material deposition within renal tubules. Evidence of nephrotoxic action includes the binding of these toxins to kidney tissues as planted antigens and their presence in the urine of exposed mice. Furthermore, experimental vaccination of mice with mutated isoforms of PLDs has shown protective effects against nephrotoxic damage and lethality caused by crude venoms [3,31,41,43]. In addition to hemolytic and renal alterations observed in systemic loxoscelism, thrombocytopenia in the peripheral blood is also reported. Although rare, it can complicate patient treatment. The involvement of PLDs in thrombocytopenia is supported by in vitro studies showing that PLDs induce platelet aggregation, a phenomenon triggered by PLDs from the venoms of various species [44,45,46]. Hematological studies corroborate this, as rabbits exposed to *Loxosceles intermedia* venom exhibit decreased platelet counts in the peripheral blood and depression of megakaryocytes in the bone marrow, indicating a correlation between precursor cells and decreased platelet levels. This condition is reversible, with platelet counts returning to normal a few days after toxin exposure [47].

Finally, the action of venom components on platelets is reflected in the hematological parameters of patients that presented thrombocytopenia [48]. In addition to the cutaneous and systemic alterations mentioned above, the literature describes other clinical changes, including those affecting cardiac and hepatic functions, which are also attributed to PLDs. However, these changes are rarely observed in clinical settings and/or confined to experimental and laboratory studies. Mice exposed to recombinant PLD from *L. intermedia* exhibited alterations in cardiac function, including increased levels of creatine kinase in the blood, and demonstrated binding of PLDs as planted antigens in cardiac tissue. Additionally, cardiomyocytes isolated from these animals showed changes in calcium influxes [49]. The potential for venom toxins to induce myocarditis was further supported by a clinical case in which a patient displayed electrocardiogram abnormalities and elevated levels of cardiac markers, including type B natriuretic peptide, troponin I, and creatine kinase [50]. Furthermore, rats exposed to either crude venom from *L. intermedia* or purified recombinant PLD under laboratory conditions exhibited changes in liver function. This was evidenced by histopathological alterations, including tumefacted hepatocytes and apoptotic cells. The animals also showed increased levels of biochemical markers of hepatotoxicity, such as gamma-glutamyl transferase (gamma GT) and transaminases [51].

## 4. Biological Activities of Brown Spider Venom Phospholipases D on Endothelial Cells

The toxic effects of *Loxosceles* venom PLDs in cutaneous and systemic loxoscelism clearly reflect their biological activities on various cell types. Among the harmful activities of the venom involving PLDs, the most extensively studied and documented in the literature is their ability to induce tissue necrosis, particularly affecting the epidermal and dermal structures (as previously discussed). It is no surprise that these PLDs are also referred to as ‘dermonecrotic toxins’. Over years of research, scientific literature has demonstrated that the dermonecrosis at the bite site and surrounding areas, along with the characteristic gravitational spreading of the lesion described in cutaneous loxoscelism, histopathologically identified as ‘aseptic coagulative necrosis’, reflects an unregulated inflammatory response in both injured patients and laboratory-tested animals [53]. Endothelial cells in the dermal blood vessels are key targets for PLDs and contribute to the exacerbated and unregulated inflammatory response seen in loxoscelism, which is associated with aseptic necrosis. Activated endothelial cells, in turn, interact with leukocytes, particularly neutrophils, leading to heterophilic adhesion between endothelial cells and leukocytes within blood vessels. This interaction triggers diapedesis, migration through the extracellular matrix, and destruction of surrounding tissue structures, including vessels, the extracellular matrix, the dermis, epidermis, and sometimes even muscle tissue in the hypodermis. These events have been well-documented through histopathological analyses of the skin of rabbits exposed to venoms from various species and purified PLDs [31,53]. All these tissue destructions contribute to generalized necrosis of the skin at the bite site and surrounding areas, leading to pain, edema, erythema, local hemorrhage, gravitational spreading of the lesion, and a blackened eschar at the site, as discussed above. The initial connection between *Loxosceles* venom toxins and endothelial cells is evident in the ability of venom toxins to bind to the surface of endothelial cells in culture, inducing morphological changes consistent with cell activation, such as increased membrane projections [54]. The binding of PLDs to the endothelial cell surface was demonstrated using a rabbit aorta cell line (RAEC) [55] treated with recombinant PLD from *L. intermedia* venom and confirmed by labeling the toxin on the cell surface with fluorescent probes [56]. Furthermore, the sensitivity of endothelial cells to *Loxosceles* venom toxins has been demonstrated over the years. Endothelial cells exposed to *L. reclusa* venom show increased expression of surface E-selectin, as well as elevated expression and secretion of leukocyte-activating cytokines such as interleukin-8 (IL-8) and granulocyte/macrophage-colony stimulating factor (GM-CSF) [57]. Additionally, the exposure of human endothelial cells to *L. deserta* venom resulted in increased expression and secretion of chemokines such as monocyte chemoattractant protein-1 (MCP-1), growth-regulated oncogene-alpha (Gro-α), and nuclear factor kappa B (NF-κB) [58,59]. 

Undoubtedly, recent advancements in understanding the cellular biology of brown spider venom toxins have been significantly enhanced by the application of various recombinant isoforms of wild-type and mutated PLDs from different species (as previously discussed in [2]). In this context, experiments using recombinant PLDs have complemented the data obtained from crude venoms or pre-purified fractions. The ability of recombinant PLDs to bind, modulate, and activate endothelial cells was demonstrated using a recombinant PLD from *L. intermedia* venom. When exposed to cultured blood vessel endothelial cells, this PLD bound to the cell surface, degraded surface phospholipids, and stimulated ectosome formation. Additionally, it triggered the intracellular activation of mRNAs involved in leukocyte activation and migration, such as Interleukin-8 [30,56]. Notably, early studies using crude venom from *L. reclusa* or a fraction purified with sphingomyelinase D activity demonstrated a direct inhibitory effect on cultured leukocytes, blocking neutrophil migration and chemotaxis [60]. All these experimental data reveal that toxins in venoms, particularly PLDs, act as agonists on endothelial cells, stimulating them to produce and secrete bioactive molecules like cytokines and chemokines. These molecules, in turn, act on leukocytes, triggering an indirect and unregulated inflammatory response.

## 5. Biological Activities of Brown Spider Venom Phospholipases D on Fibroblasts and Keratinocytes

**Figure 2:**
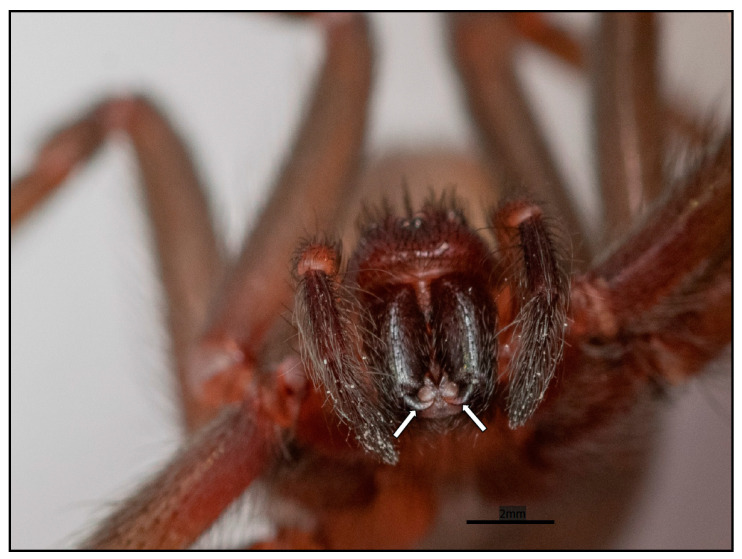
Macroscopic view of the chelicerae of *Loxosceles intermedia*. Photomicrograph captured with a Nikon D5300, Speedlight SB 700, and Sigma 105 mm macro lens, showing two hook-shaped chelicerae (white arrows) at the venom-injecting apparatus. Scale bar: 2 mm.

Fibroblasts and keratinocytes are two additional skin cell types that can be targeted by PLDs. Fibroblasts, located in the dermis where venom is injected via the chelicerae, represent another cell type sensitive to PLDs. Research has demonstrated that PLDs can influence fibroblasts, leading to the production of biological mediators that indirectly activate leukocytes and trigger an uncontrolled inflammatory response, which contributes to the pathophysiology of cutaneous loxoscelism (as previously discussed). Studies of human fibroblasts in culture revealed that treatment with recombinant PLD from *L. reclusa* venom led to increased gene expression of various cytokines and chemokines involved in the inflammatory response, including IL-6, IL-8, IL-1β, CCL5, CXCL1, CXCL2, and tumor necrosis factor-α. These findings suggest that the bioactive molecules secreted by fibroblasts, in conjunction with those from endothelial cells, may contribute to the activation of leukocytes and the unregulated inflammatory response observed in cutaneous loxoscelism [61]. Skin fibroblasts treated with PLDs from *L. laeta* have been shown to increase the expression and secretion of cytokines and chemokines such as IL-6, IL-8, CXCL1, and CCL2. The authors suggest that the interaction of PLDs with fibroblasts trigger signaling cascades that activate nuclear factor kappa-beta, a key regulator of the inflammatory response as described in the literature. Once again, it is speculated that fibroblasts may work in concert with endothelial cells to drive the inflammatory response observed in the cutaneous form of loxoscelism [62]. Another potential target for PLDs in the skin is keratinocytes. These cells, located in the epidermis, are separated from the dermis by a basement membrane (extracellular matrix), creating two distinct compartments. Based on the size and organization of the chelicerae (the venom-injecting organ) (see Figure 2), it is believed that venoms and toxins are injected into the dermis. Consequently, there is no direct exposure of toxins to keratinocytes; however, toxins may indirectly reach and activate these cells through diffusion. Studies using cell cultures demonstrated the effects of venom components and PLDs on keratinocytes. Pioneering research from 2000 revealed that human keratinocytes exposed to crude venom from *L. reclusa* exhibited increased expression of vascular endothelial growth factor (VEGF), indicating sensitivity to the treatment [63]. VEGF is a molecule involved in angiogenesis and vascular permeability and may contribute to the activation of endothelial cells. This activation, in turn, can stimulate leukocyte activity, which, as previously discussed, plays a role in dermonecrosis and skin injuries. Studies of human keratinocytes exposed to recombinant PLD from *L. laeta* venom demonstrated that the treatment induced cell death by apoptosis. According to the authors, this process is dependent on the expression and secretion of extracellular matrix metalloproteases MMP2, MMP7, and MMP9. Treatment with tetracycline, an MMP inhibitor, resulted in reduced cell death and decreased expression of MMPs following exposure to PLD [64,65]. Furthermore, human keratinocytes exposed to crude venom or recombinant PLD from *L. laeta* exhibited increased intracellular production of superoxide and DNA damage, which the authors attribute to the binding of PLD to the cell surface. The data also indicated that exposure to PLD stimulated cellular DNA repair through the activation of an apparent ATR-mediated DNA-damage response. The inhibition of ATR-kinase during PLD exposure resulted in decreased cell viability, indicating the kinase’s role in keratinocyte survival following PLD treatment [66]. In addition, the exposure of human keratinocytes in culture to recombinant PLD from *L. laeta* venom triggered the activation of metalloproteases ADAM-10 and ADAM-17 on the cell surface. This activation is mediated by the serine protease furin, which acts as a convertase to transform inactive forms of ADAMs into their active forms. The authors also observed the co-localization of PLD with GM1 gangliosides found in lipid rafts, along with a decrease in caveolin-1 concentration and an increase in flotillin-1. These findings indicate that exposure to PLD induced changes in lipid rafts within the cell membranes [67].

## 6. Biological Activities of Brown Spider Venom Phospholipases D on Erythrocytes and Platelets

Red blood cells are also highly sensitive to PLDs. Erythrocytes undergo lysis upon in vitro exposure to these enzymes, which aligns to intravascular hemolysis and hemolytic anemia observed in systemic loxoscelism and often associated with complications in the clinical presentation of affected patients (as discussed earlier, see [3]). The concept that components of *Loxosceles* spider venoms induce erythrocyte lysis dates back to the early studies of PLD in brown spider venom. As early as 1978, it was reported that a native fraction from *L. reclusa* venom, characterized as sphingomyelinase D due to its activity on sphingomyelin, was capable of causing lysis in human and sheep red blood cells [17]. This lytic activity of PLDs on erythrocytes was subsequently confirmed using recombinant PLD isoforms from various species, which demonstrated significant hemolytic activity on human erythrocytes [2,8,22,42,68]. Two experimental findings highlight the hemolytic action of PLDs. First, the hemolytic activity is dependent on the catalytic function of the enzymes, as recombinant isoforms with reduced catalytic activity also exhibited reduced hemolytic effects [18]. Second, the hemolytic effect varies with the red blood cell source; human, sheep, and rabbit erythrocytes are highly sensitive to hemolysis, whereas horse erythrocytes show greater resistance, indicating that the biochemical composition of cell membranes may influence hemolytic sensitivity [42]. Another blood cell component sensitive to PLDs is platelets, which are fragments of megakaryocytes. PLDs induce platelet aggregation in platelet-rich plasma, an effect that may contribute to complications in cutaneous loxoscelism. This activity was noted in early studies describing PLDs in venoms. As early as 1981, it was demonstrated that a purified fraction of crude *L. reclusa* venom with sphingomyelinase D activity could induce the aggregation of human platelets [44]. Subsequent studies demonstrated that a purified fraction of crude *L. reclusa* venom with sphingomyelinase D activity could activate platelets in vitro, leading to serotonin release and platelet aggregation [45]. These findings regarding PLD activity on platelet activation were further supported by studies using recombinant PLD forms, which also induced platelet aggregation. It is noteworthy that platelet aggregation is dependent on the catalytic activity of PLDs, as isoforms lacking phospholipase D activity also do not affect platelets [69]. Additionally, this platelet-aggregating effect is conserved across PLDs from different venoms [2,8,46,68,69].

## 7. Biological Activities of Brown Spider Venom Phospholipases D on Kidney Cells

There are many examples of other cells that bind to and are affected by PLDs from *Loxosceles* spider venoms, but from a pathophysiological perspective, kidney cells are particularly notable targets of PLD binding and warrant special mention. Such cytotoxicity may be linked to systemic loxoscelism in its renal form, characterized by acute renal failure observed in accidents, which is associated with severe clinical complications and potentially death [1,32,34]. The involvement of PLDs in renal lesions is evidenced by alterations in renal function observed in laboratory animal models, such as mice and rats. Exposure to PLDs in these models resulted in proteinuria, hemoglobinuria, hematuria, and changes in biochemical markers like urea and creatinine. Histological analyses of animals exposed to PLDs, as well as patients, revealed changes such as glomerular edema, tubular necrosis, and the deposition of proteinaceous materials in the renal tubules, even leading to animal mortality [43,70,71]. Immunofluorescence analyses of kidneys from mice showed the binding of PLDs to cells in the glomeruli and renal tubules, where they act as ‘planted antigens’ following envenomation. These bound PLDs are subsequently excreted in the urine, which can be detected by immunoblotting with anti-PLD antibodies [43]. Another evidence for the direct action of PLDs on renal structures is the absence of an inflammatory response in the renal tissue, which rules out mechanisms similar to those observed in cutaneous loxoscelism [43]. The direct action of PLDs on the kidneys was demonstrated by the binding of toxins to MDCK (Madin-Darby Canine Kidney) epithelial cells, triggering cytotoxicity signs including morphological changes, cell bubble formation, reduced cell spreading, intercellular separation, detachment of the extracellular matrix, and decreasing cell viability [43]. HK-2 tubular epithelial cells exposed to recombinant PLD from *L. laeta* venom suffered cytotoxic effects, characterized by apoptosis and a decrease in cell viability [71]. These in vivo and in vitro experimental data indicate the involvement of PLDs in renal lesions and suggest that nephrotoxic and cytotoxic activities are dependent on catalytic activity. Recombinant isoforms mutated in the catalytic site or other regions involved in biochemical activity did not cause renal alterations in animals or MDCK cells [43].

## 8. Biological Activities of Brown Spider Venom Phospholipases D on Mast and Tumor Cells

Lastly, it is worth noting that a recombinant PLD from the venom of *Loxosceles intermedia* binds to malignant mouse cells (B16-F10 melanoma lineage) without inducing cytotoxicity or morphological changes. However, it increases cytoplasmic calcium influx and cell proliferation [72], resembling the action of Autotaxin, a phospholipase D secreted by melanoma cells. Autotaxin enhances proliferation, migration, and metastatic potential in melanoma cells [73]. Mast cells are another example of cells affected by PLDs, leading to changes with clinical and pathological significance. The role of PLDs and mast cells in the pathophysiology of envenomation was first suggested when mice were inoculated with recombinant PLD from *L. intermedia* venom on their paws or back. The evaluation revealed edematogenic activity and increased vascular permeability in a dose- and time-dependent manner. The observation that pre-treatment of animals with the drug 48/80 (a pharmacological agent that stimulates mast cell degranulation) significantly inhibited edema and vascular permeability highlighted the involvement of mast cells in these events [74]. Similarly, treatment with the histamine H1 receptor antagonists promethazine and cetirizine, as well as the serotonin receptor antagonist methysergide, reduced the edematogenic activity and vascular permeability induced by PLD, further supporting the involvement of mast cells in these processes [74]. In addition, the necessity of PLD functionality and catalysis for edema and vascular permeability was demonstrated, as enzyme denaturation by heating and a recombinant isoform mutated at the catalytic site (H12A) were unable to induce edema or vascular permeability [74]. However, there is no evidence of direct actions of PLDs on mast cells. On the contrary, all attempts to demonstrate the direct activity of a wild-type recombinant PLD isoform from *L. intermedia* in vitro on RBL-2H3 cells, a widely accepted mast cell model, failed to show degranulating activity [74]. This suggests that PLD may act synergistically with other venom toxins or stimulate indirect processes that ultimately lead to mast cell degranulation. In this context, two molecules from *Loxosceles* venoms have been identified, cloned, and produced in recombinant form. These include LiTCTP, a Translationally Controlled Tumor Protein (TCTP) family member identified in *L. intermedia* venom, which also functions as a histamine-releasing factor, and LALT (*Loxosceles* Allergen-Like Toxin) [3], both of which have shown direct potential to degranulate RBL-2H3 cells and mouse mesentery mast cells. Moreover, the involvement of mast cells in cutaneous loxoscelism is supported by experimental data showing that mice subcutaneously implanted with polyester–polyurethane disks and subsequently exposed to the crude venom of *L. similis* exhibited mast cell infiltration and degranulation in the implant, as confirmed by histological analysis [75].

## 9. Pathophysiology of Envenoming Based on the Functional Activities of PLDs on Target Cells, Plasma Membranes, Ectosome Formation, and Ectocytosis

**Figure 3:**
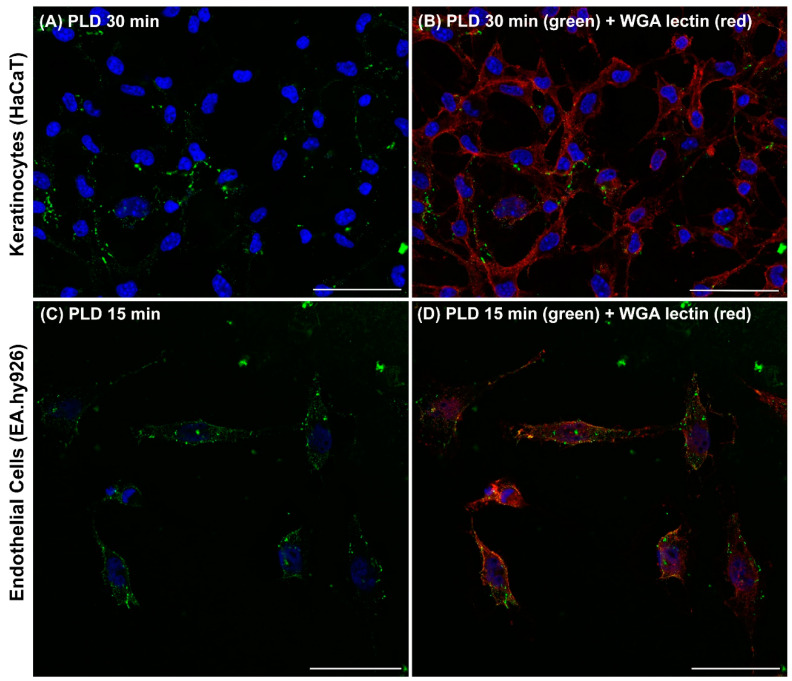
Recombinant PLD from *Loxosceles intermedia* venom binds to the surface of human endothelial (EA.hy926) and keratinocyte (HaCaT) cells, stimulating the formation of extracellular microvesicles. Human EA.hy926 endothelial cells were incubated with recombinant PLD for 15 min, and HaCaT keratinocyte cells for 30 min, under culture conditions at 37 °C. After incubation, cells were washed with phosphate buffer, fixed with formaldehyde, and labeled with an anti-PLD antibody (green), Wheat Germ Agglutinin (red) to mark the cell surface, or DAPI (blue) to stain nuclei. Confocal fluorescence microscopy revealed PLD binding to the cell surface and the formation of extracellular microvesicles budding from the plasma membrane into the extracellular environment. These microvesicles, characterized as ectosomes and associated with ectocytosis (see Justa et al. [30]), were imaged using a Nikon A1R MP+ multiphoton confocal microscope at 600× magnification. Scale bars: 50 μm.

Given the size and structural organization of the chelicerae, which resemble hypodermic hooks involved in venom injection, it is expected that the venom is delivered directly into the dermis of the injured individual (Figure 2). The chelicerae measure approximately 240 μm, while the distance from the surface of the skin (stratum corneum) to the beginning of the dermis, just below the basement membrane that separates the epidermis from the dermis, ranges from 76.9 ± 26.2 μm to 267.4 ± 120.6 μm [76]. Therefore, the chelicerae are sufficiently large to penetrate the epidermis and deliver venom into the dermis, reaching the cells located there. Hence, it is expected that cells residing in the dermis, such as fibroblasts and blood vessel cells like endothelial cells—which are sensitive to the actions of PLDs, as previously mentioned—can be affected by venom inoculation. Consequently, these cells may become targets for the toxins, contributing to the pathogenesis of cutaneous loxoscelism. Neutrophils within blood vessels and keratinocytes in the epidermis (separated from the dermis by a basement membrane) can be indirectly activated by toxins in the venom, as well as by cytokines, chemokines, or membrane degradation products—such as biologically active lipids—released by cells directly affected by PLDs. Thus, a proposed cellular mechanism for cutaneous loxoscelism involves the activation of endothelial cells by the direct action of PLDs: these enzymes bind to the plasma membrane and release lipid fragments derived from surface phospholipids, which could theoretically act as autocrine signals within the endothelium, triggering cascades of intracellular signaling. Additionally, products generated after PLD activity can function as paracrine signals, affecting distant cells such as leukocytes—particularly neutrophils—which play a central role in cutaneous loxoscelism. These cells are activated and contribute to the uncontrolled inflammatory response discussed earlier in this text. An interesting observation is that the binding of PLDs to the surface of endothelial cells stimulates the production of microvesicles or ectosomes from the plasma membrane. These microvesicles are released into the extracellular environment through ectocytosis, carrying PLDs and lipid/protein components from the cell membrane, and can thus act at a distance as paracrine signals [30,77]. This binding to endothelial cells does not appear to depend on amino acid residues involved in the catalysis of phospholipid degradation, such as Histidines H12 and H47, as a PLD isoform with mutations (H12A-H47A) binds to the surface of endothelial cells similarly to the wild-type forms [30]. However, PLDs with mutations at Tryptophan W230 (W230A)—an amino acid that, along with Y228 and Y229, is crucial for interactions with phospholipids via choline and cation–π bonds [31,32]—or mutations at residues E32/D34 (E32A-D34A)—important for coordinating the magnesium ion, which appears to form an intermediate complex with the PLD and phospholipid substrates [2,25,26,28]—exhibit significantly reduced binding to the surface of endothelial cells and decreased ectosome formation compared to wild-type forms [30]. The involvement of different amino acid residues in various regions of PLDs, which play a role in binding to the cell surface, suggests that these enzymes interact with cell membranes through multiple binding sites. This observation aligns with the spatial organization of these PLDs, which resemble an (α/β)8 barrel with an expanded surface area [8,25,26], enabling the PLDs to interact with membrane ligands through various binding sites. Another interesting observation is that, despite their reduced binding to the surface of endothelial cells and lack of catalytic activity in phospholipid degradation, isoforms of mutated PLDs (W230A or E32A-D34A) still induce cellular responses when exposed to endothelial cells. This activation can be evidenced by the generation of microvesicles or ectosomes on the cell surface, indicating supramolecular changes in the membranes [30]. Additionally, an increase in mRNA expression of Interleukin-8 in cultured endothelial cells, as well as the activation of an inflammatory response in vivo, occurs when these mutated PLD isoforms are injected into the skin of rabbits. This phenomenon, as discussed earlier, is associated with skin necrosis. However, unlike the effects of wild-type PLD isoforms, the mutated PLD isoforms do not induce dermonecrosis or progress in leukocyte infiltration, suggesting that the necessary molecular conditions for intracellular or extracellular activation are not met [30]. These data offer an explanation for previously unexplained results in the literature, which show that recombinant PLD isoforms with mutations in various amino acid residues—despite being inactive from a biochemical and functional perspective—can still induce cell activation with cytokine and chemokine production upon exposure to cultured cells [23]. Thus, it appears that the binding of PLDs to the cell surface alone is sufficient to disrupt cellular processes, leading to the production of microvesicles/ectosomes, alterations in the supramolecular organization of cell membranes, and the initiation of intracellular signaling. Finally, the secretion of extracellular microvesicles through ectocytosis signalosomes can activate other cells involved in the pathophysiology of loxoscelism, such as leukocytes, keratinocytes, fibroblasts, erythrocytes, and platelets, among others. This process helps to explain the noxious events described. However, as noted by Justa et al. [30], after binding to the cell surface, the catalytic activity of PLDs in degrading phospholipids from the cell membrane—resulting in the production of bioactive molecules such as ceramide-1-phosphate, lysophosphatidic acid, or their cyclic forms like ceramide-1,3-phosphate (as discussed earlier)—is essential for the progression of these initial cellular signals and the intense inflammatory response associated with dermonecrosis. Another interesting observation is that the generation of microvesicles/ectosomes on the surface of cells exposed to PLDs aligns with preliminary data showing the supramolecular organization of synthetic membranes exposed to a recombinant PLD from *Loxosceles laeta* venom [78]. Additionally, the formation of microvesicles/ectosomes in cells exposed to PLDs may act as a mechanism for removing agents that disturb the plasma membrane, as cells treated with recombinant PLDs, even at high concentrations, do not internalize these PLDs into acidic intracellular compartments such as endosomes or lysosomes nor do they exhibit signs of cytotoxicity [30]. This behavior is also consistent with previously observed effects of other agents involved in the formation of microvesicles [79,80]. It is also interesting that the data show that endothelial cells fixed and processed for histological analysis—despite having denatured membrane components and undergoing cell death—continue to bind PLDs. In this case, however, microvesicle/ectosome formation does not occur; instead, there is a linear fluorescent labeling on the cell surface [30]. This suggests that PLD binding to cells is independent of protein conformation, as the cell surfaces were denatured by histological fixation procedures. These data strongly suggest that the binding of PLDs to the cell surface depends on plasma membrane lipids rather than proteins, which are more susceptible to denaturation during fixation. Therefore, it appears that the pathophysiology of PLDs causing dermonecrosis in affected patients depends on the direct binding of the enzymes to lipids on the surface of target cells, such as endothelial cells in blood vessels. This binding alone leads to the formation of microvesicles/ectosomes, which are secreted into the extracellular environment through ectocytosis. Additionally, it triggers the intracellular activation of endothelial cells, resulting in the production of transcripts for signaling molecules involved in initiating the inflammatory response. Ectocytosis produces paracrine or autocrine signalosomes that disseminate signaling molecules to neighboring cells, such as keratinocytes, and primarily to polymorphonuclear leukocytes, which play a central role in the aseptic necrotizing process associated with cutaneous loxoscelism, driven by an uncontrolled inflammatory response as previously discussed [2,8]. The mechanism of interaction between PLDs and the surface of target cells depends on the metal magnesium [30]. As discussed earlier in this text, magnesium is a component of PLDs, making them metalloenzymes. Mutated PLD isoforms that are impaired in magnesium coordination bind less effectively to the surface of target cells, such as endothelial cells, and also produce fewer ectosomes through ectocytosis. Additionally, these mutated isoforms stimulate a reduced inflammatory response in vivo [30]. All these data align with the findings in the literature indicating that bivalent metals like magnesium can form a bridge between a metalloprotein and the cell surface through electrostatic interactions, leading to the binding of the metalloprotein as a peripheral protein on the cell membrane [81]. Crystallographic data of recombinant PLDs also highlight the role of the magnesium ion in stabilizing the interaction between PLDs and phospholipid substrates during degrading catalysis [25,26,82], reinforcing the idea that magnesium is crucial for the interaction with lipids on the surface of target cells. Additionally, the formation of extracellular microvesicles/ectosomes following the exposure of cells to PLDs is noteworthy as a conserved and biologically significant event, as endothelial cells treated with recombinant PLDs from the venoms of *L. intermedia*, *L. laeta*, and *L. gaucho* all produced ectosomes [30]. It is noteworthy that the microvesicles formed and secreted into the extracellular environment are coated with PLDs, allowing these enzymes to travel with the vesicles and reach distant cells. This enhances the potential for signal propagation due to the actions of PLDs on the endothelium and supports the occurrence of systemic loxoscelism. This includes effects on other cells such as erythrocytes (causing hemolysis and hemolytic anemia), platelets (leading to thrombocytopenia), and renal tissue (resulting in acute renal failure), as previously discussed [2,3,8]. It is also worth noting that, in addition to cultured rabbit aorta endothelial cells (RAEC), human endothelial cells, keratinocytes (Figure 3), and fibroblasts exposed to recombinant PLDs also produce ectosomes and ectocytosis [30]. This indicates that PLD activities are not limited to the endothelium but can occur in various cell types.

## 10. Future Perspectives

**Figure 4:**
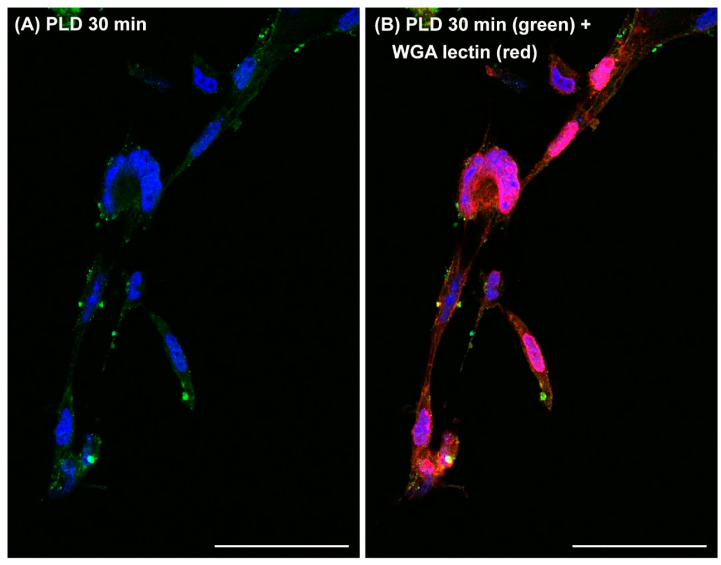
Recombinant PLD from *Loxosceles intermedia* venom binds to B16-F1 melanoma murine cells, inducing extracellular microvesicles and supramolecular structure formation. B16-F1 melanoma cells were incubated with 40 μg of recombinant PLD for 30 min under culture conditions at 37 °C. After incubation, cells were washed with phosphate-buffered saline, fixed with formaldehyde-containing buffer, and labeled with an anti-PLD antibody (green), Wheat Germ Agglutinin (red) to mark the cell surface, or DAPI (blue) to stain nuclei. Tumor cells exposed to recombinant PLD displayed granular fluorescence on the surface, along with ectosome formation on the plasma membrane, leading to the development of supramolecular structures budding into the extracellular environment. Images were captured using a Nikon A1R MP+ multiphoton confocal microscope at 600× magnification. Scale bars: 50 μm.

The finding that cells exposed to PLDs from *Loxosceles* spider venoms produce microvesicles/ectosomes and ectocytosis—and that these events are involved in loxoscelism—opens up extensive possibilities for understanding the underlying cellular mechanisms and exploring biotechnological applications of these toxins. The understanding of how these PLDs affect different cells is now more directed, given that the cellular biology of ectosomes and ectocytosis is already reasonably well understood [83]. If the pathophysiological events resulting from PLD actions on various cells depend on the formation of ectosomes and ectocytosis, it opens up possibilities for testing inhibitors as potential treatments for cutaneous and systemic loxoscelism. This would be the first time in the literature describing a rational approach to apply different drugs for treating loxoscelism based on the activity of a venom component, as current treatments rely on serum therapy or symptomatic pharmacology. The literature describes several inhibitors of microvesicle formation [84], which could be tested for their ability to mitigate injuries caused by these toxins following accidents. Another application of this knowledge is in the laboratory diagnosis of loxoscelism. Currently, there is no reproducible and standardized laboratory method for diagnosing this condition. The diagnosis is based on capturing the spider, which rarely occurs, and on whether the patient comes from an area endemic to spiders and related accidents. This includes regions in South America, such as southern Brazil, and parts of Argentina, Peru, and Chile, where loxoscelism is a significant public health issue [2,3,8]. The formation and systemic spread of ectosomes containing PLDs on their surfaces can be used to detect these microvesicles in patient secretions and biological fluids. By applying concentration methods such as ultracentrifugation combined with immunostaining, this approach provides a reliable and reproducible method for diagnosing loxoscelism. Finally, the discovery that different cells treated with PLDs produce ectosomes and ectocytosis could form the basis for a method to extract these cellular structures from various cells, including tumor cells (see Figure 4). This procedure creates a straightforward and reproducible method for studying these important cellular structures as signalosomes, opening new possibilities for understanding the biological and pathological roles of extracellular microvesicles in the cellular biology of both normal and malignant cells. With an emphasis on the potential for transferring biomolecules (lipids, proteins, and nucleic acids) from donor microvesicle cells to recipient cells, thereby regulating physiological and/or pathological events [85]. One of the major challenges in oncology is that tumor cells often evade detection by the immune system. Tumor markers and antigens may be ‘hidden’ from immune cells, or tumors may secrete molecules that inhibit the body’s defenses, making it difficult for the immune system to recognize and eliminate these cells [86,87,88]. Ectosomes formed after treating various tumor cells with Brown spider venom PLDs can carry tumor components that are relevant for immune system activation, expose hidden antigenic markers, or transport inhibitory molecules. If these components are recognized by immune cells, they could enhance the body’s defense against tumors. This presents significant possibilities for improving defense cell activation in vitro through exposure to ectosomes or for developing anti-tumor vaccines. The literature indicates that ectosomes derived from tumor cells carry fragments of cell membranes, along with nucleic acids and secreted molecules. These ectosomes play a significant role in tumor cell biology, as signals in cellular communication, tumor growth, and resistance to anti-tumor drugs [89]. PLD-induced tumor ectosomes and ectocytosis could serve as a model where tumor cells, typically adept at evading detection, begin to communicate in a molecular language that might be better understood by immune cells. This model could be valuable for studying tumor growth inhibition or strategies to block tumor spread.

## Data Availability

The original contributions presented in this study are included in the article. Further inquiries can be directed to the corresponding author.
